# Immunological assay using a solid-state pore with a low limit of detection

**DOI:** 10.1038/s41598-024-67112-8

**Published:** 2024-07-19

**Authors:** Hiroyasu Takei, Tomoko Nakada, lat Wai Leong, Atsuki Ito, Kakeru Hanada, Hinako Maeda, Muhammad Shan Sohail, Kazuhiko Tomiyasu, Osamu Sakamoto, Norihiko Naono, Masateru Taniguchi

**Affiliations:** 1 Aipore Inc., 26-1 Sakuragaokacho, Shibuya, Tokyo 150-8512 Japan; 2 SANKEN, Osaka University, 8-1 Mihogaoka, Ibaraki, Osaka 567-0047 Japan

**Keywords:** Nanopores, Biosensors, Nanopores

## Abstract

Emerging infectious diseases, cancer, and other diseases are quickly tested mainly via immune reactions based on specific molecular recognition between antigens and antibodies. By changing the diameter of solid-state pores, biomolecules of various sizes can be rapidly detected at the single-molecule level. The combination of immunoreactions and solid-state pores paves the way for an efficient testing method with high specificity and sensitivity. The challenge in developing this method is achieving quantitative analysis using solid-state pores. Here, we demonstrate a method with a low limit of detection for testing tumor markers using a combination of immunoreactions and solid-state pore technology. Quantitative analysis of the mixing ratio of two and three beads with different diameters was achieved with an error rate of up to 4.7%. The hybrid solid-state pore and immunoreaction methods with prostate-specific antigen (PSA) and anti-PSA antibody-modified beads achieved a detection limit of 24.9 fM PSA in 30 min. The hybrid solid-state pore and immunoreaction enabled the rapid development of easy-to-use tests with lower limit of detection and greater throughput than commercially available immunoassay for point-of-care testing.

## Introduction

Solid-state pores fabricated on solid substrates with a thickness of < 50 nm can detect and identify analytes individually depending on the diameter of the solid-state pore^1–5^. Solid-state pores with a diameter of a few nm can detect and identify DNA^4,6–10^, RNA^11,12^, and proteins^13–16^. Solid-state pores with diameters of several 100 nm and several µm can detect and identify viruses^17–21^ and bacteria^22,23^, respectively. The great attraction of solid-state pores is that they can detect and identify wide-scale biomolecules at the single-molecule level without chemical modification of the solid-state pore, without using molecular recognition ability. However, not using molecules with molecular recognition ability is a hurdle to obtaining high selectivity in detection and identification.

An antigen–antibody reaction (an immunoreaction), a prime example of precise molecular recognition capability, is a well-established principle of enzyme-linked immunosorbent assay (ELISA) and immunoassay methods for diagnosing infectious diseases and cancer^24–26^. The combination of antigens, antibodies, and beads can be used to develop the desired test method. Optimizing this combination can control the sensitivity and specificity of the test. The ability of the immune reaction to recognize molecules controls the selectivity of the detection and identification of the target analyte. If the immune reaction can be detected and discriminated at the single-molecule level, a highly sensitive and specific test can be developed.

The combination of a solid-state pore, which can detect and discriminate at the single-molecule level, and an immune reaction with high selectivity can be developed into an innovative testing method^27,28^ with improved adaptability to the detection target (adaptive pore: “AdaPore”). In this study, we have developed AdaPore, which combines solid-state nanopores and immunoreaction using antibody-modified nanoparticles. To confirm that AdaPore is capable of antigen quantification, the dependence of pulse frequency on nanoparticle size and concentration, applied voltage, and hydraulic pressure was investigated. We found that solid-state nanopores with a diameter of 1.3 µm can be used for quantitative analysis of nanoparticles with a diameter of 250 nm or larger. The measurement and analysis of mixed solutions of two or three nanoparticles with different diameters demonstrated that quantitative analysis of the mixing ratio is possible. Using solid-state nanopores with a diameter of 1.3 µm and antibody-modified nanoparticles with a diameter of 300 nm, we found that PSA could be detected down to 24.9 fM. This limit of detection is approximately 1000 times greater than that of commercially available immunoassays point-of-care testing. Moreover, it is possible to develop a testing system with a low limit of detection by selecting the appropriate nanoparticles for the targeted antigen–antibody reaction as AdaPore is adaptable to all immune reactions.

## Results

### Adapore system

**Figure 1:**
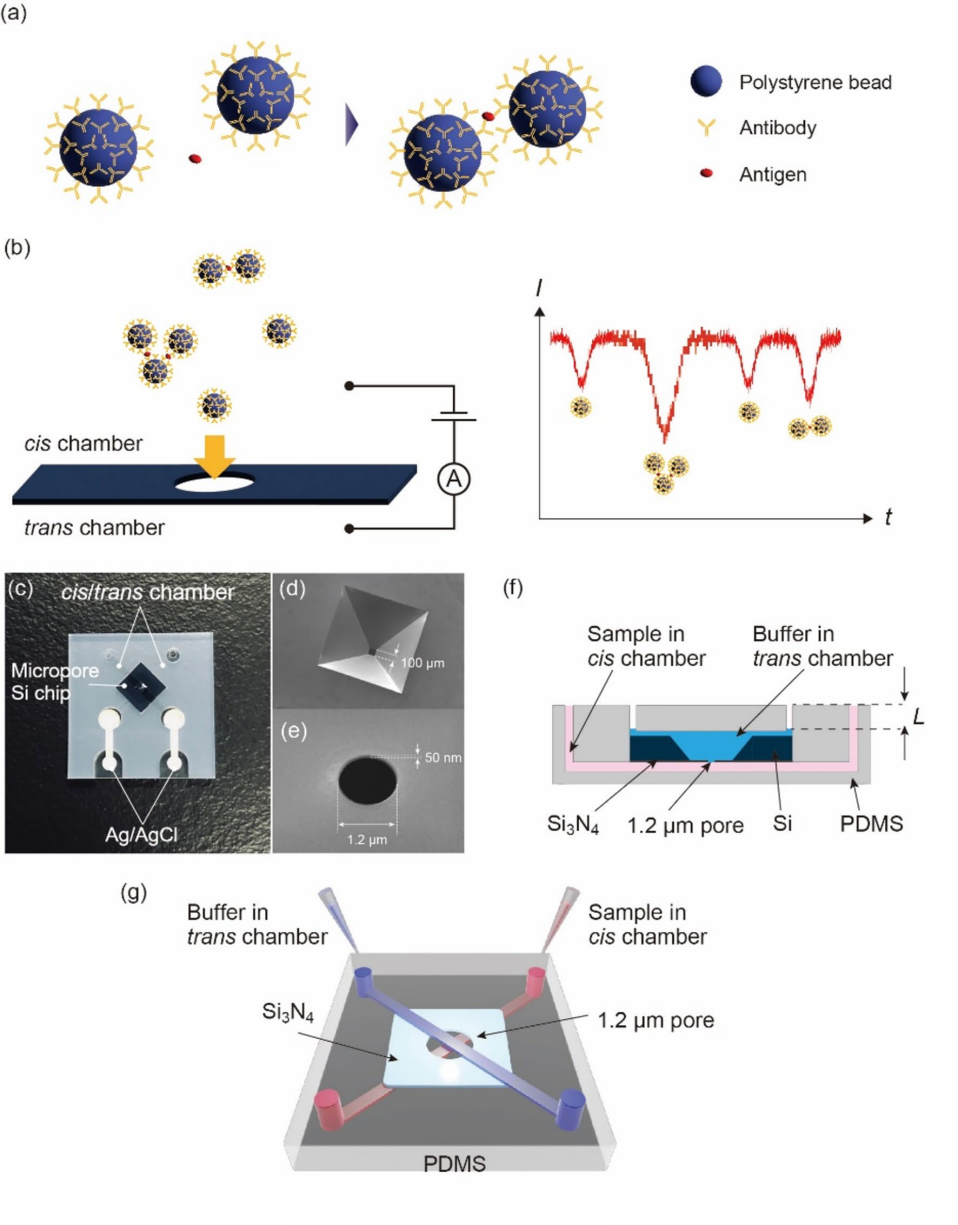
Adapore system. (**a**) Antibody-modified polystyrene beads are dispersed in a solution. When an antigen is added to the aqueous solution, aggregates of polystyrene beads are formed by the antigen–antibody reaction. (**b**) Isolated antibody-modified polystyrene beads and aggregates flow from the top to the bottom due to hydrostatic pressure and electrophoresis. The change in the ion current depends on the degree of aggregation of the beads as they pass through the solid-state pore. Large aggregates provide large changes in the ionic current. (**c**) The solid-state pore module used in the experiment. The black area in the center is the silicon substrate on which the solid-state pore is fabricated. The Ag/AgCl electrodes were fabricated by printing. The *cis* and *trans* channels fabricated by PDMS have a crossed spatial arrangement. (**d**) Only the center part is dug by wet etching to hold the Si_3_N_4_ thin film, which is 100-μm square and 50-nm thick. (**e**) SEM image of a solid-state pore with a diameter of 1.2 μm and a length of 50 nm at a 40° tilt angle. (**f**) Cross-sectional view of a solid-state pore module in which the hydraulic pressure can be controlled by the amount of liquid in the *cis* chamber. By adjusting the liquid volume in the *cis* chamber, *L* is adjusted to control the hydraulic pressure. (**g**) 3D schematic of the solid-state pore module. Corresponding to the 2D cross-sectional view shown in Fig. 1f, the *cis* and *trans* chambers are shown in red and blue, respectively.

The Adapore system consists of an antigen, antibody-modified polystyrene beads, a solid-state pore module, an ion current analyzer, and analysis software. Polystyrene beads modified with two types of antibodies are dispersed in an aqueous solution. An antigen–antibody reaction aggregates the beads when antibodies are added to the aqueous solution (Fig. 1a). The higher the amount of the antibody, the greater the degree of aggregation. This method is a one-step reaction in which the antigen and antibody-modified beads are simply mixed.

When the *cis* and *trans* chambers of the solid-state pore module are filled with 1× PBS and voltage is applied, an ion current flows between the Ag/AgCl electrodes. When a mixture of the antigen and antibody-modified beads is added to the *cis* chamber, monodispersed negatively charged and aggregated antibody-modified beads are transported into the *trans* chamber. The higher the degree of aggregation, the greater the change in the ion current (Fig. 1b).

The driving force for analyte transport in most solid-state pores is electrophoresis due to the voltage between the electrodes and electroosmotic flow generated on the charged solid-state pore walls^29–31^. If a higher voltage is applied to obtain more ionic current–time waveforms per unit of time, waveforms will not be obtained because the analyte passes through the solid pore too quickly^32–34^. One approach for obtaining many ionic current–time waveforms per unit of time is introducing a voltage-independent driving force^8,35–38^. Here, using hydraulic pressure as a driving force for the analyte, we have developed a solid-state pore module that can change the height of the aqueous solution in the *cis* and *trans* chambers (Fig. 1c–g).

A solid-state pore with a diameter of 1.2 μm was fabricated on 50 nm-thick silicon nitride deposited on a 5-mm square silicon wafer. Then, 1× PBS at volumes of 18 and 15 µL were added to the *cis* and *trans* chambers, respectively. Because of the higher solution height in the *cis* chamber, the direction of the water flow due to hydraulic pressure was from the *cis* chamber to the *trans* chamber (Fig. 1f,g). The ion current measured at the sampling rate of 250 kHz was 182 ± 27 nA at 0.1 V. Under conditions without hydraulic pressure with 1× PBS at 15 µL in both the *cis* and *trans* chambers, the ion current was 201 ± 17 nA at 0.1 V. Using the resistance equation 4*hρ*/π*d*^2^ + *ρ*/*d* for the solid-state pore, we obtained an ion current of 183 nA^39,40^. *h*, *d*, and *ρ* are the thickness and diameter of the solid-state pore and the ionic conductivity at 1× PBS, respectively. The experimentally obtained ion current values are within the error range and are comparable to theoretical values. Thus, under these experimental conditions, hydraulic pressure does not affect the ionic current.

### Single-bead measurements

**Figure 2:**
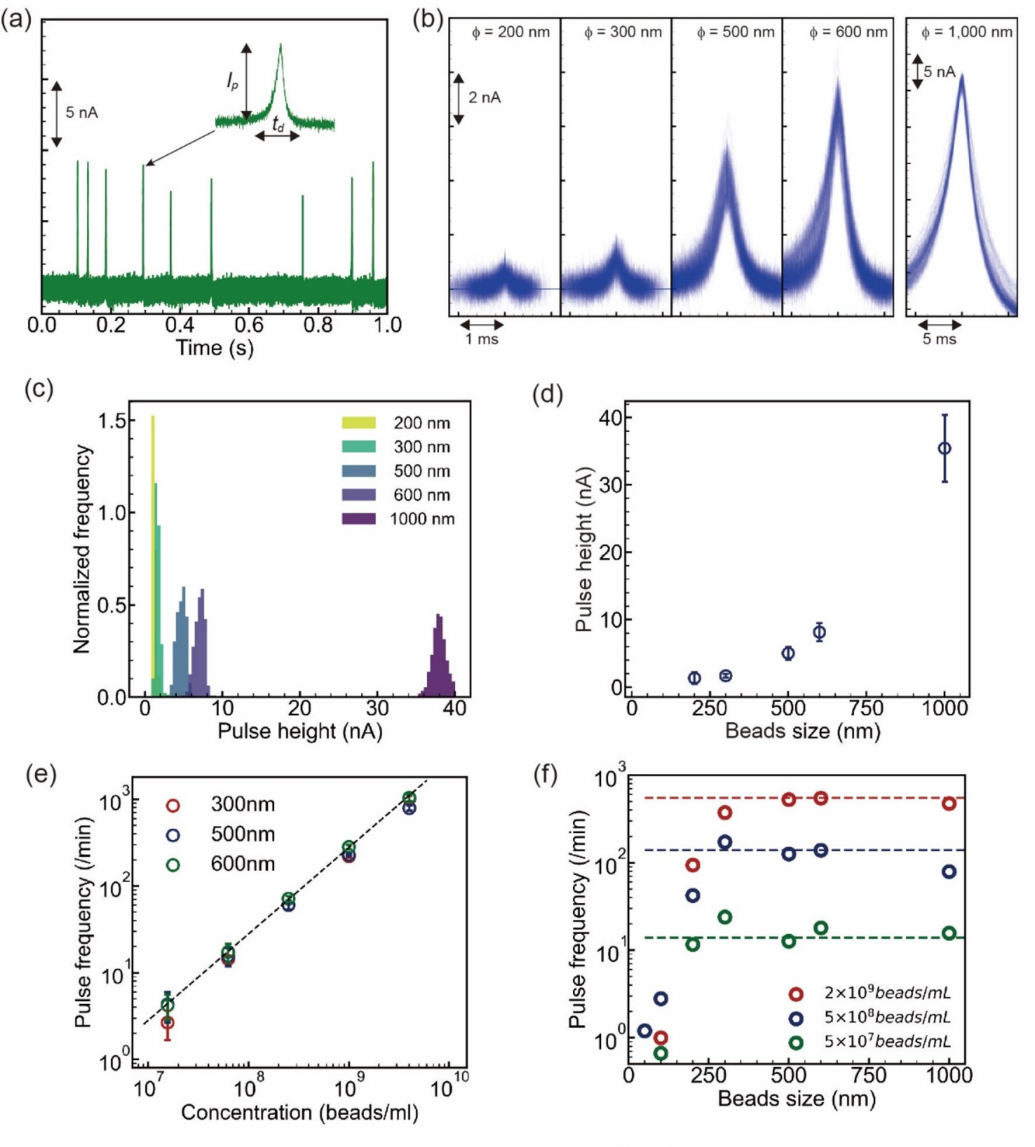
Measurements of polystyrene beads transported by hydraulic pressure and electrophoresis. (**a**) Ionic current–time profile of 600-nm polystyrene beads obtained by solid-state pore measurement. The vertical axis is the amount of change in decreasing ion current. *I*_p_ and *t*_d_ indicate the maximum current value and current duration, respectively. (**b**) Ionic current–time waveforms of 100 polystyrene beads with different diameters (*ϕ*). (**c**) Bead size dependence of the *I*_p_ histogram. (**d**) The *I*_p_ histogram of mode current versus bead size. More than 1000 waveforms were obtained for each bead size. Error bars represent 1× SD. (**e**) Dependence of pulse frequency on bead concentration. Polystyrene beads with diameters of 300, 500, and 600 nm were measured with three solid pore modules, respectively. Error bars represent 1× SD. (**f**) Bead size dependence on pulse frequency. Red, blue, and green indicate bead concentrations of 2 × 10^9^ beads/mL, 5 × 10^8^ beads/mL, and 5 × 10^7^ beads/mL, respectively. The three colored dotted lines indicate the saturation pulse frequency at each bead concentration.

Polystyrene beads with diameters of 200 nm, 300 nm, 500 nm, 600 nm, and 1 μm were suspended in 1× PBS with 0.05% Tween-20. The *cis* and *trans* chambers were filled with 18-µL bead suspension and 15-µL 1× PBS, respectively. The applied voltage was 0.1 V. When one bead passed through the solid-state pore, a spike-like ionic current–time pulse was obtained (Fig. 2a). The pulse is characterized by two factors: the peak current *I*_p_ and the current duration *t*_d_. The commercial software Aipore-ONE™^20^ was used to extract the pulses and calculate the *I*_p_ and *t*_d_.

The larger the diameter of the beads, the higher the *I*_p_ and the more uniform the pulse shape (Fig. 2b and Table S1). The smaller the bead diameter, the larger the ion current–time pulse dispersion due to the off-axis effect^41–43^. The histograms of the *I*_p_ showed that the distributions of *I*_p_ differ depending on the size of the bead (Fig. 2c). The smaller the beads, the smaller the dispersion of the *I*_p_. The mode frequency of the *I*_p_ histogram for each bead size had a squared relationship with the bead size (Fig. 2d)^40^. The obtained equation for the relationship between the mode current and bead size indicates that measurements can be performed down to a diameter of 250 nm. The pulse frequency per minute obtained for different concentrations of beads with diameters of 300 nm, 500 nm, and 600 nm showed a proportional relationship to the concentration (Fig. 2e and Table S2). We found that the pulse frequency was independent of the bead size when the bead concentration was the same. To further clarify the relationship between pulse frequency, concentration, and bead size, the dependence of pulse frequency on bead size at three concentrations (5 × 10^7^, 5 × 10^8^, and 2 × 10^9^ beads/mL) was investigated (Fig. 2f and Table S3). The pulse frequency was independent of the bead size for the same concentrations and bead sizes > 250 nm. Beads with a diameter of < 250 nm passed through the solid-state pore; however, the ion current change was too small to be detected because it was buried in the electrical noise. The observation that the pulse frequency is independent of the bead size but depends on the bead concentration makes it possible to quantitatively determine the bead concentration from the pulse frequency. Furthermore, the quantitative analysis of the mixing ratio of two or more beads with different bead sizes becomes possible.

### Quantitative analysis of the mixing ratio of two types of beads with different diameters

Because one pulse obtained by solid-state pore measurement corresponds to one bead, the relationship of the number of pulses to the number of beads is valid. Therefore, solid-state pore measurement has a counting function that counts beads individually. When two types of beads with different diameters exist in a solution, the mixing ratio of the two types of beads can be determined by the count of pulses for each bead. A mixed solution of beads with diameters of 300 nm and 500 nm was measured in a solid-state pore. If the counts of all beads and the counts of beads with a diameter of 500 nm are *N* and *k*, respectively, the mixing ratio of beads with a diameter of 500 nm can be estimated by *k*/*N*.

A 1× PBS suspension of beads with diameters of 300 and 500 nm was prepared at a concentration of 4 × 10^9^ beads/mL. Mixing solutions were prepared with mixing ratios of 0%, 1.0%, 2.5%, 5.0%, 10.0%, 20.0%, and 40.0% for a diameter of 500 nm. Then, 18-µL mixed solution and 15-µL buffer solution were placed in the *cis* and *trans* chambers, respectively. Each mixture was measured with three solid-state pore modules with a voltage of 0.1 V (Table S4).

**Figure 3:**
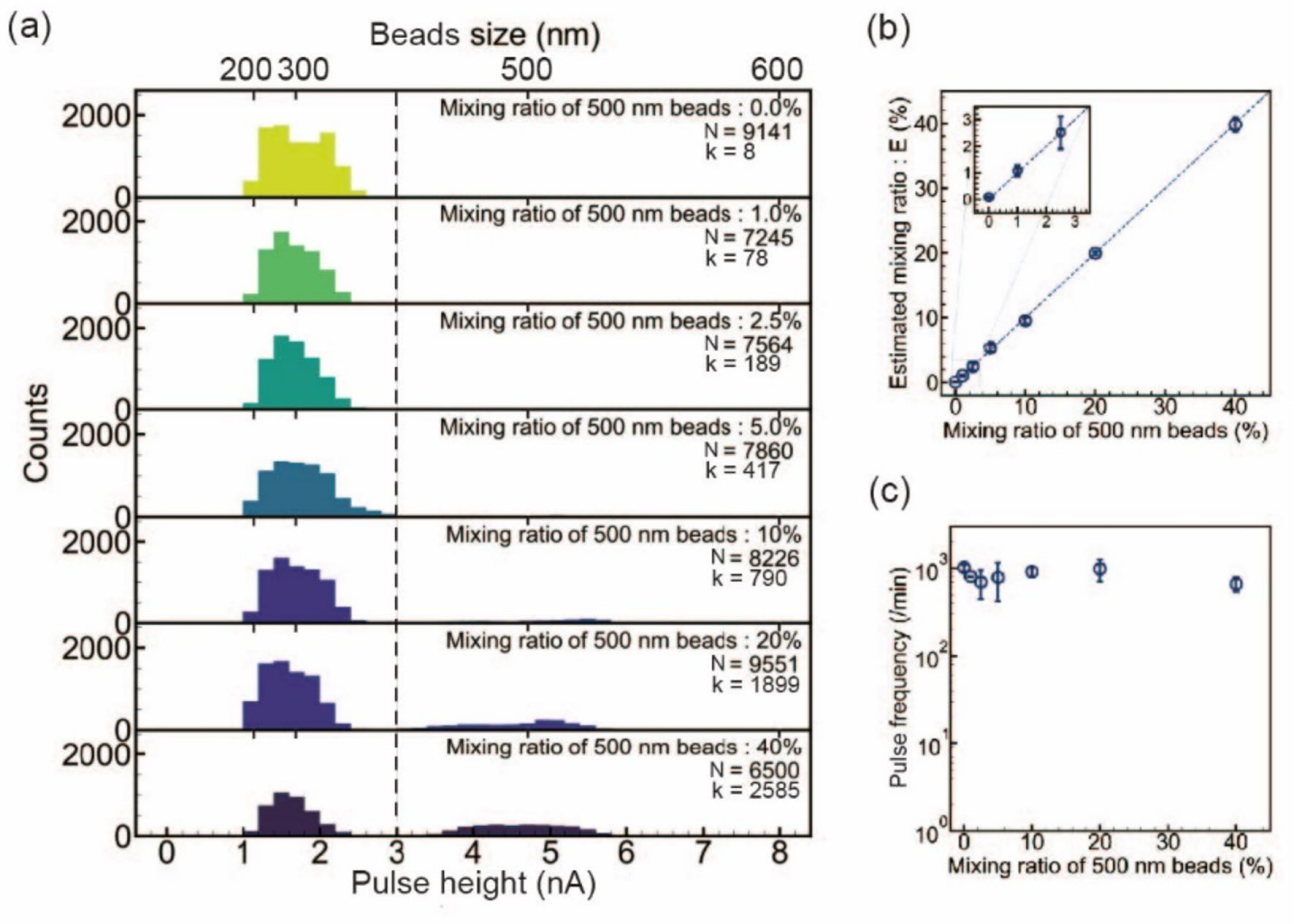
Quantitative analysis of mixed solutions with different mixing ratios of beads with diameters of 300 and 500 nm. (**a**) Pulse height (*I*_*p*_) histogram obtained by measuring mixed solutions with different mixing ratios of beads with diameters of 300 and 500 nm. The bead concentration was adjusted to 4 × 10^9^ beads/mL. Measurements at each mixing ratio were made using three solid-state pore modules. The dotted line indicates the threshold (3 nA) that distinguishes 300-nm-diameter beads from 500-nm-diameter beads. *N* and *k* denote the total number of pulses obtained in the measurement and the number of pulses attributed to 500-nm-diameter beads, respectively. (**b**) Relationship between the mixing ratio of 500-nm-diameter beads estimated by the solid-state pore measurement and the actual mixing ratio. Error bars indicate 1× SD. (**c**) Correlation between the mixing ratio of the beads and pulse frequency. Error bars indicate 1× SD.

*I*_p_ histograms of the pulses obtained from the measurement of the 0% mixture solution showed only dispersion corresponding to a bead size of 300 nm (Fig. 3a). As the mixing ratio of beads with a diameter of 500 nm increased, the dispersion corresponding to beads with a diameter of 500 nm became clearly observed. The observation of two types of beads with different diameters was also evident from the two-dimensional distribution of *I*_p_ − *t*_d_ (Fig. S1). Measurement of a suspension containing nanoparticles with two diameters may yield current–time waveforms indicative of the simultaneous translocation of two types of nanoparticles. When two types of nanoparticles pass simultaneously through the solid-state pore, I_p_ and t_d_ values larger than those observed for nanoparticles with a diameter of 500 nm are expected. In particular, the fraction of waveforms exhibiting I_p_ ≥ 6 nA or t_d_ ≥ 5 ms constituted only 0.25% of the total waveforms (Fig. S1g). This indicates that the likelihood of simultaneous translocation of the two types of nanoparticles is considerably low. From the *I*_p_ histograms of the beads with diameters of 300 and 500 nm, the *I*_p_ values below and above 3 nA were attributed to beads with diameters of 300 and 500 nm, respectively (Fig. 2c). The counts of beads with diameters of 300 and 500 nm were obtained from the histograms. The mixing ratio (*k*/*N*) of beads with a diameter of 500 nm showed a perfect proportional relationship with the actual mixing ratio (Fig. 3b). The pulse frequency was independent of the mixing ratio of beads with a diameter of 500 nm (Fig. 3c).

### Quantitative analysis of the mixing ratio of three types of beads with different diameters

**Figure 4:**
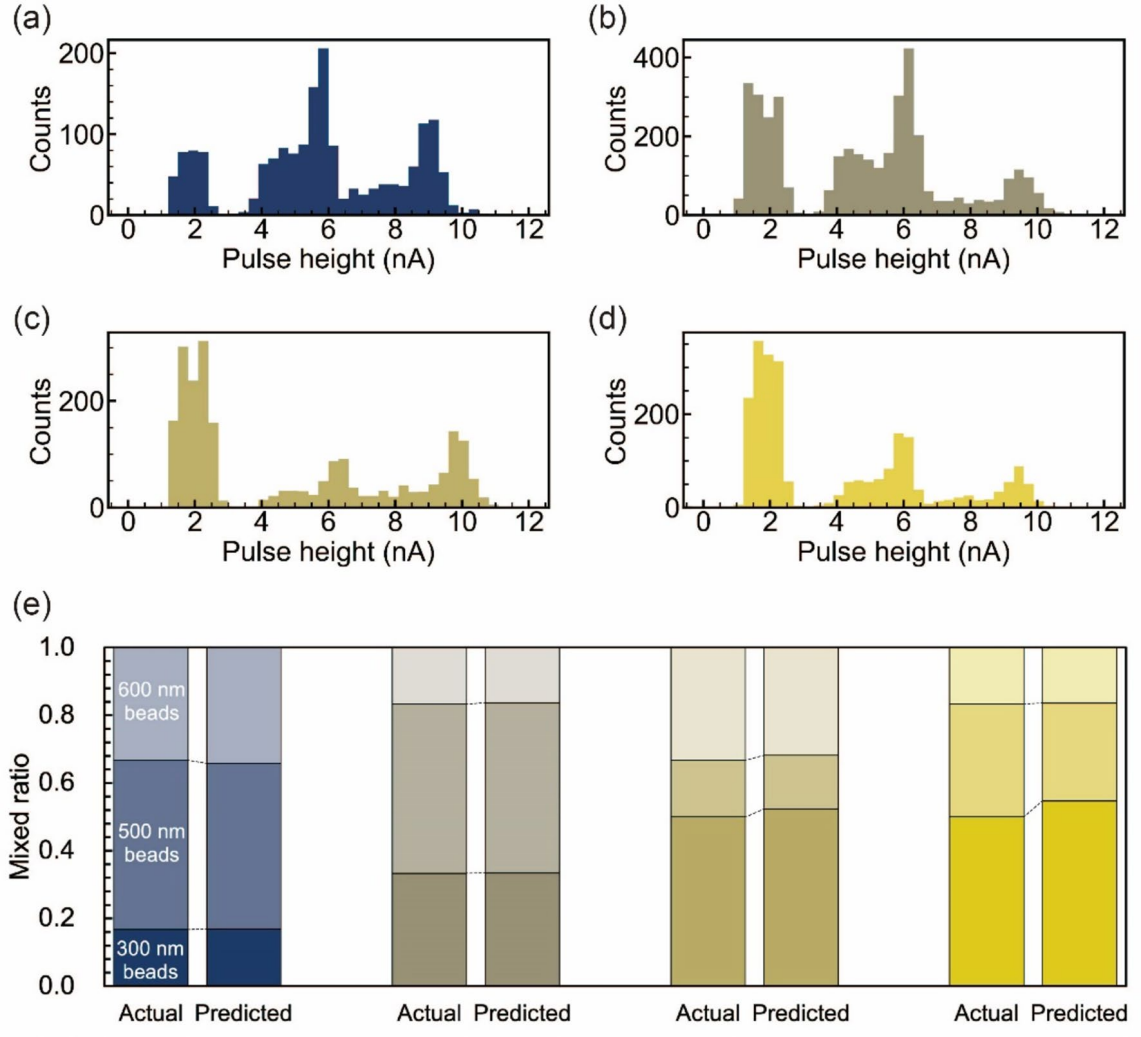
Quantitative analysis of mixed solutions of polystyrene beads with diameters of 300, 500, and 600 nm at different mixing ratios. Pulse height (*I*_p_) histograms obtained by solid-state pore measurement of solutions mixed in the ratio 300 nm:500 nm:600 nm at (**a**) 1:3:2, (**b**) 2:3:1, (**c**) 3:1:2, (**d**) 3:2:1. (**e**) Comparison between the mixing ratio estimated by solid-state pore measurement and the actual mixing ratio: 300 nm:500 nm:600 nm. The color codes of the *I*_p_ histograms match the color codes of the ratio bars.

Quantitative analysis of mixing ratios based on counting was adapted to a mixed solution of three types of beads with different diameters. Then, 1× PBS suspension solutions with diameters of 300, 500, and 600 nm were prepared at a concentration of 1 × 10^9^ beads/mL. A mixture of the three types of beads was prepared at a mixing ratio of 1:3:2 (Fig. S2). The *I*_p_ histogram for each pulse obtained via measurements using the solid-state pore module showed three peak current values (Fig. 4a). Furthermore, measurement of suspensions containing nanoparticles with three diameters may yield waveforms corresponding to the simultaneous translocation of two or three types of nanoparticles. When such simultaneous passages occur, I_p_ and t_d_ are anticipated to be greater than those for nanoparticles with a diameter of 600 nm. The fraction of waveforms with I_p_ ≥ 10 nA or t_d_ ≥ 7 ms was 0.40% of the total waveforms (Fig. S2), indicating the minimal probability of such simultaneous translocations. Referring to the *I*_p_ histograms of the three beads (Fig. 2c), the threshold values of the currents attributed to the three beads was set to 3.0 and 5.9 nA. The mixing ratio obtained from the number of pulses attributed to the three beads was 300 nm:500 nm:600 nm = 1:3:2 (Fig. 4a and Table S5). Similarly, mixed solutions were prepared with mixing ratios of 300 nm:500 nm:600 nm = 2:3:1 (Fig. 4b and Table S5), 300 nm:500 nm:600 nm = 3:1:2 (Fig. 4c and Table S5), and 300 nm:500 nm:600 nm = 3:2:1 (Fig. 4d and Table S5) for the three bead types. The ratios were calculated by counting the number of pulses. The average and maximum error ratios for the mixing ratio of each bead calculated from counting the number of pulses were 2.0% and 4.7%, respectively (Fig. 4e). The mixing ratios calculated from measuring mixed solutions with mixing ratios of 50.0%, 33.3%, and 16.7% were 54.7%, 28.9%, and 16.4%, respectively. The maximum error rate was 54.7% − 50.0% = 4.7%. This result indicates that the quantitative analysis of the mixing ratio of three different beads with different diameters is feasible.

### Quantitative analysis of prostate-specific antigen (PSA)

Antibody-modified beads with a diameter of 300 nm are aggregated by an antigen–antibody reaction. Here, PSA, a commonly used marker for prostate cancer, was used as an antigen^44^. Then, 1× PBS, PSA, and anti-PSA antibody-modified polystyrene beads were mixed and incubated at room temperature for 20 min. Next, 18 µL of the mixed sample and 15-µL 1× PBS were placed in the *cis* and *trans* chambers, respectively. A voltage of 0.1 V was applied, and measurements were taken for 10 min. The PSA concentration was adjusted to 29.4 pM (1 ng/mL), 2.94 pM (100 pg/mL), 0.294 pM (10 pg/mL), 29.4 fM (1 pg/mL), 2.94 fM (100 fg/mL), and 0 M (Fig. S3 and Table S6).

**Figure 5:**
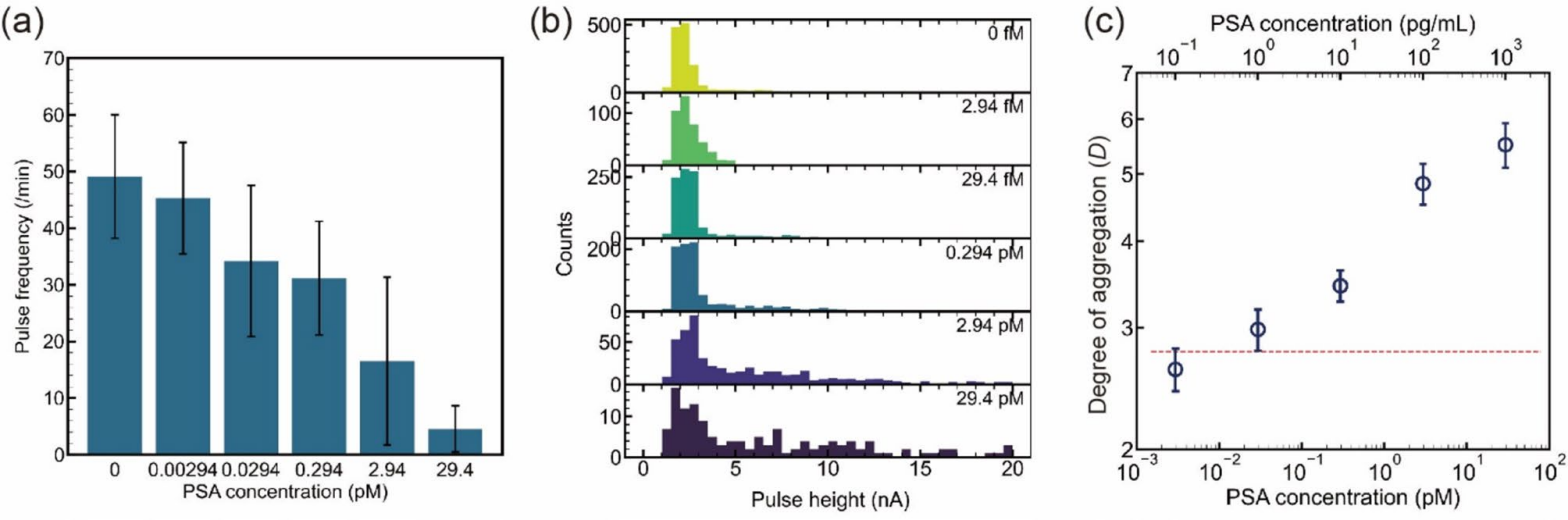
Quantitative analysis of PSA using Adapore. Polystyrene beads were modified with an anti-PSA antibody. (**a**) Dependence of pulse frequency on PSA concentration. Error bars indicate SD. (**b**) Dependence of the pulse height (*I*_p_) histogram of the ionic current–time waveform on PSA concentration. Each concentration was measured using three solid-state pore modules. (**c**) Dependence of aggregation on PSA concentration. For each concentration, measurements were made using three solid-state pore modules. Error bars indicate ± 3 SD. The dotted line denotes the mean ± 3 SD of the negative control (PSA concentration: 0 fM).

Quantitative analysis of an antigen is accomplished when the concentration of antibody-modified beads and aggregates is between 10^7^ and 10^10^ beads/mL (Fig. 2e). The initial concentration of antibody-modified beads was estimated to be 3.7 × 10^8^ beads/mL based on the optical density value. The average pulse frequencies obtained when 29.4-pM, 2.94-pM, 0.294-pM, 29.4-fM, 2.94-fM, and 0-fM PSA were added were 4.5 min^−1^, 16.5 min^−1^, 31.1 min^−1^, 34.2 min^−1^, 45.2 min^−1^, and 49.1 min^−1^, respectively (Fig. 5a). Each pulse frequency corresponds to a bead concentration of 5.1 × 10^7^ beads/mL, 9.8 × 10^7^ beads/mL, 1.5 × 10^8^ beads/mL, 1.7 × 10^8^ beads/mL, 2.1 × 10^8^ beads/mL, and 2.3 × 10^8^ beads/mL, respectively (Fig. 2e). These results indicate that the number of pulses is proportional to the bead concentration for quantitative analysis over the full concentration range of PSA used in the experiment.

The *I*_p_ histogram of the pulses obtained from the measurements showed a maximum frequency of 1.5 nA, corresponding to beads with a diameter of 300 nm, for all concentrations of PSA (Fig. 5b). This indicates that many beads do not aggregate. When 0.294-pM PSA was added, the *I*_p_ histograms showed peaks at 1.5, 2.8, 3.5, and 6.0 nA (Fig. S4). The 2.8-, 3.5-, and 6.0-nA peaks could correspond to aggregates of 2, 3, and 4 beads, respectively. Even without PSA, few pulses above 3.0 nA were detected. This is due to the self-aggregation of the antibody-modified beads. As the concentration of PSA increased, the number of pulses observed above 3 nA increased. Distinguishing whether the aggregates are due to the antigen–antibody reaction or self-aggregation is difficult. Here, the degree of dispersion of the histogram was used to evaluate the degree of aggregation (*D*) (the definition of *D* is given in Supporting Information). The *D* and standard deviation of the PSA at 0 M were 2.64 and 6.62 × 10^−2^, respectively (Fig. 5c). *D* values above 2.64 indicate the formation of aggregates due to the antigen–antibody reaction. The relationship between *D* and the PSA concentration indicates that the limit of detection is 29.4 fM.

To evaluate the Adapore method’s performance with clinical specimens, we conducted tests using pseudoclinical samples prepared by spiking PSA into fetal bovine serum. Despite optimizing various parameters such as incubation time, surfactant concentration, filter mesh size for pretreatment, and blocking agents to minimize nonspecific reactions, we encountered challenges with nonspecific aggregation when using the PSA antibody (Fig. S5). This nonspecific aggregation elevated the aggregation ratio and led to pore clogging, even in negative control samples, rendering the determination of the limit of detection for pseudoclinical samples inconclusive. It is evident that further optimization of the PSA antibodies is essential for the successful application of Adapore to clinical samples.

## Discussion

**Figure 6:**
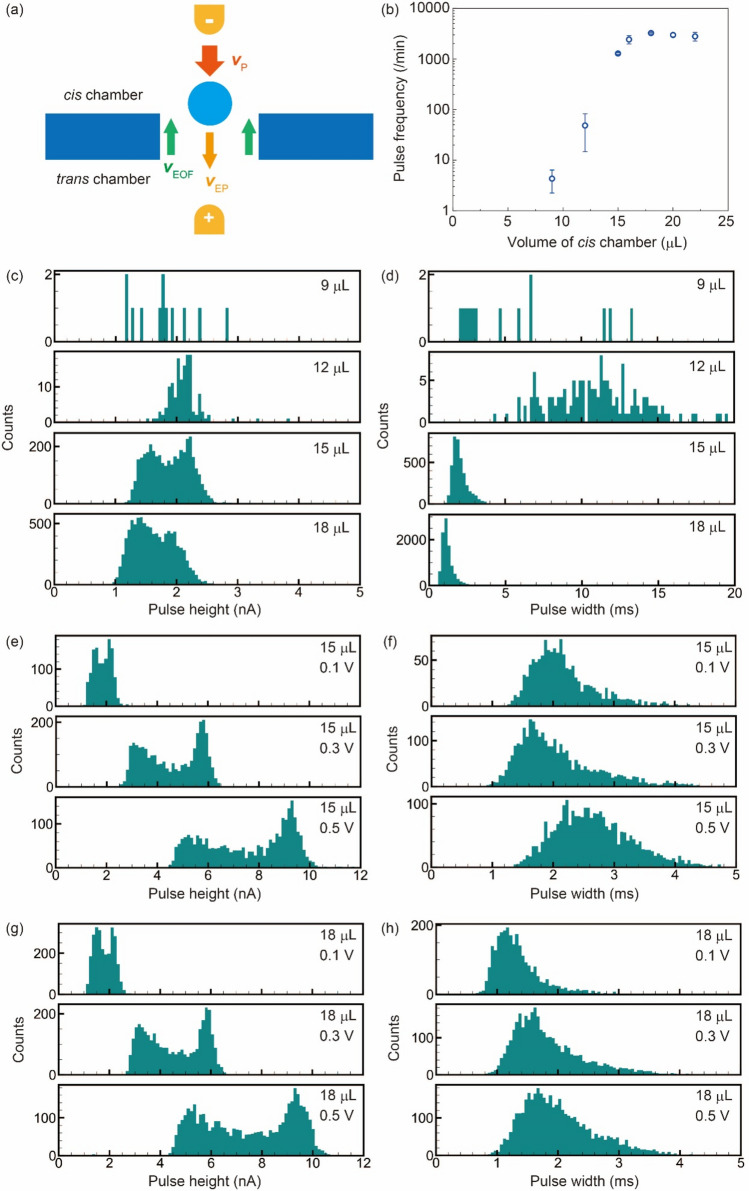
Flow dynamics of nanoparticles. (**a**) The three flows that contribute to the flow dynamics of nanoparticles when hydraulic pressure is applied to the *cis* chamber side. Negatively charged nanoparticles are subjected to electrophoresis and hydraulic pressure in the downward direction from the top. The direction of the electroosmotic flow is from the bottom to the top because cations accumulate at the solid–liquid interface of solid-state pores with negative surface charges. (**b**) Dependence of pulse frequecy. The error bars indicate ± 3 SD. Sample volume dependence of (**c**) pulse height (*I*_p_) and (**d**) pulse width (*t*_d_) at 0.1 V. Voltage dependence of (**e**) *I*_p_ and (**f**) *t*_d_ for a sample volume of 15 μL. Voltage dependence of (**g**) *I*_p_ and (**h**) *t*_d_ for a sample volume of 18 μL. For all pore measurements, the *trans* chamber volume was 15 μL.

The flow dynamics of nanoparticles are affected by electrophoresis (*v*_EP_), electroosmotic flow (*v*_EO_), and hydraulic pressure (*v*_P_) (Fig. 6a)^30,31^. Nanoparticles with a negative surface charge are subjected to forces from the *cis* chamber toward the *trans* chamber due to electrophoresis. Cations that accumulate at the solid–liquid interface of SiN with a negative surface charge are subjected to a force from the *trans* chamber toward the *cis* chamber, which generates an electroosmotic flow. The hydraulic pressure applied to the *cis* chamber causes the liquid to flow from the *cis* chamber toward the *trans* chamber. First, to confirm the transport of beads by hydraulic pressure, at sample volumes of 15 and 18 μL, the surface of a solid-state pore in the 0 V state was observed using optical microscopy from the *trans* chamber side (Supporting Videos 1 and 2). At the sample volume of 15 μL, no beads were observed passing through the solid-state pore. At the sample volume of 18 μL, beads transported from the *cis* chamber into the *trans* chamber were observed with high frequency. This observation demonstrates that hydraulic pressure can transport beads.

To test the effect of hydraulic pressure under the application of 0.1 V, solid-state pore measurements were performed using different sample volumes in the *cis* chamber using a 300-nm nanoparticle. As the sample volume decreased, the pulse frequency decreased drastically (Fig. 6b). At sample volumes of 9 and 12 μL, the pulse frequency decreased because the solution flowed from the *trans* chamber to the *cis* chamber. At sample volumes < 15 µL, the direction of the bead transport by hydraulic pressure was opposite to that by electrophoresis. When the sample volume was 15 μL, the pulse frequency was 1280 ± 50 (1/min). When the sample volume was 16, 18, 20, and 22 μL, the pulse frequencies were 2420 ± 449, 3237 ± 104, 2977 ± 251, and 2791 ± 545 (1/min), respectively. Pulse frequencies ranged from 1.9 to 2.5 times the pulse frequency at 15 μL and appeared to be saturated above 18 μL. This result indicates that hydraulic pressure increases pulse frequency.

The sample volume dependence of *I*_p_ and *t*_d_ was then investigated (Fig. 6c,d). For sample volumes of 9 and 12 μL, the number of pulses was not statistically sufficient. At a sample volume of 15 μL, the histogram of *I*_p_ showed two modes, 1.6 and 2.1 nA. The peak current at 2.1 nA was due to the off-axis effect of nanoparticles flowing near the pore wall^41–43^. When the sample volume was increased to 18 μL, the peaks in the histogram of *I*_p_ decreased to 1.3 and 1.8 nA. The two *t*_d_ modes at sample volumes of 15 and 18 μL were 1.8 and 1.1 ms, respectively. This result indicates that hydraulic pressure accelerates the translocation speed of nanoparticles. One of the reasons for the decrease in *I*_p_ when hydraulic pressure is applied is the change in the base current.

The dependence of the base current on the sample volume was measured. The base current obtained from the measurements was 152 nA ± 4 and was independent of the sample volume. At a sample volume of 18 μL, the difference between the water surface heights of the *cis* and *trans* chambers was *L* = 3 mm. This difference in height gave a hydraulic pressure of approximately 30 Pa. The multiphysics model simulation of the dependence of the base current on hydraulic pressure showed that the base current was 199.0 nA at sample volumes of 15 and 18 μL. Therefore, both the experiments and simulations showed that the base currents at 15 and 18 μL were the same. To further investigate the variation of *I*_p_ with hydraulic pressure, simulations of a steady-state model with nanoparticles placed at the center of solid-state pores were performed at 0 and 30 Pa. The ion currents at 0 and 30 Pa were both 197.8 nA. The *I*_p_ was 199.0 − 197.8 = 1.2 nA, which is comparable to the experimental value. Therefore, the experimental and simulation results for the base current indicate that the difference between the two experimental modes of *I*_p_ was due to experimental errors.

Multiphysics model simulations showed that fluid velocities at the pore center at 0 and 30 Pa were in opposite directions (Fig. S6). This result indicates that the hydraulic pressure at 30 Pa transports nanoparticles from the *cis* chamber to the *trans* chamber. Furthermore, the simulation results suggest that the effect of the electroosmotic flow directs the fluid velocity from the *trans* chamber to the *cis* chamber at 0 Pa. Therefore, to investigate the effect of electroosmotic flow, the dependence of *I*_p_ and *t*_d_ on applied voltage was examined using sample volumes of 15 and 18 μL (Fig. 6e–h). In this experiment, the histograms of *I*_p_ also showed two modes. The larger *I*_p_ was due to the off-axis effect^41–43^. For applied voltages of 0.1, 0.3, and 0.5 V, at a sample volume of 15 μL, the lower *I*_p_ mode increased to 1.5, 3.6, and 6.1 nA, respectively; the higher *I*_p_ mode increased to 2.1, 5.7, and 9.2 nA, respectively; and at a sample volume of 15 μL, the *t*_d_ mode was 2.1, 1.8, and 2.6 ms, respectively. At 0.3 V, *t*_d_ was considered to have decreased because the increase in electrophoretic flow was larger than the increase in electroosmotic flow. However, at 0.5 V, the increase in velocity due to electroosmotic flow exceeded the increase in velocity due to electrophoresis, resulting in an increase in *t*_d_. For applied voltages of 0.1, 0.3, and 0.5 V, at a sample volume of 18 μL, the lower *I*_p_ mode increased to 1.5, 3.7, and 6.0 nA, respectively, and the higher *I*_p_ mode increased to 2.1, 5.8, and 9.3 nA, respectively. The *I*_p_ modes were the same as the *I*_p_ modes at a sample volume of 15 μL. For applied voltages of 0.1, 0.3, and 0.5 V, the *t*_d_ mode increased to 1.2, 1.6, and 1.8 ms, respectively. Since the flow direction of nanoparticles due to the electrophoretic force and hydraulic pressure was the same, the increase of *t*_d_ with increasing applied voltage was caused by an increase in electroosmotic flow.

Immunoassays, particularly those utilizing antigen–antibody interactions, are integral in diagnosing a wide range of infectious diseases and cancers. This discussion primarily focuses on the PSA test, a staple in clinical laboratories worldwide^45^. The conventional method for PSA detection is the Chemiluminescent Enzyme Immunoassay (CLEIA), which, akin to ELISA, employs enzyme-labeled antigens or antibodies in a luminescent reaction. Unlike ELISA, which relies on absorbance, CLEIA measures luminescence, with a standard PSA detection threshold set between 2 and 4 ng/mL globally^45^. Among the innovations in this domain is the digital ELISA, known for its low detection limit achieved through fluorescent detection of antigens by antibody-coated nanoparticles in individual nanowells^46,47^. Its detection capability extends to 10 zM for pure samples and 0.4 fM for clinical specimens^47^. However, the scale of digital ELISA equipment limits its application in Point-of-Care Testing (POCT). Solid-state pores represent a viable alternative for POCT, offering single-molecule detection by monitoring the volume of specimens passing through the pore. This principle has been applied in detecting nanoparticle aggregates resulting from antigen–antibody reactions and DNA complementarity^28,48^. For instance, combining nanopores with electromagnets and antibody-coated magnetic nanoparticles has achieved a detection limit of 0.8 fM in blood samples^49^, and surface modification of nanopores with antibodies has further reduced this limit to 80 aM^50^. The prevailing antigen/antibody assays are, however, time-intensive. The Adapore system distinguishes itself with its compact size and swift testing process, taking just 30 min. Unlike traditional methods that rely on histograms of ion current changes, Adapore’s innovation lies in the distinct ion current–time waveforms of single nanoparticles versus aggregates. We are exploring machine learning techniques to classify these waveforms^20^, potentially enabling precise identification of individual nanoparticle aggregates and further enhancing detection sensitivity.

## Conclusion

We have developed the Adapore system that combines solid-state nanopores and immunoreaction using antibody-modified nanoparticles. The pulse frequency was proportional to the concentration of beads in the sample and independent of bead diameters > 250 nm. The use of antibody-modified beads with a diameter of 300 nm enabled the quantification of bead aggregates generated by antigen–antibody reactions. When the antigen and antibody were PSA and anti-PSA, respectively, the LOD was 29.4 fM. This LOD is 1000 times more sensitive than the LOD achieved by commercially available immunochromatography for point-of-care testing^51^. By combining the solid-state pore technology, an antigen, an antibody, and beads, the Adapore system allows the development of targeted quantitative tests.

## Methods

### The AdaPore system

A micropore sensor device (M-AS-1200-A028-001-Ai) and a current measurement device with a built-in current amplifier (MicroSCOUTER WEL1200) were purchased from Aipore Inc. Ion currents were sampled at 250 kHz.

### Polystyrene bead measurement

Polystyrene beads (Nanosphere 3000 series) were purchased from Thermo-Fisher Scientific (USA). Then, 1× PBS and Polyoxyethylene (20) sorbitan monolaurate were purchased from FUJIFILM Wako Pure Chemical Corp. (Osaka, Japan). Polyoxyethylene (20) sorbitan monolaurate was used as an alternative for Tween-20. The applied voltage during the ion current measurements was 0.1 V. Aipore-ONE™^20^ (Aipore Inc.), commercial software, was used to calculate the height *I*_p_ and width *t*_d_ of the pulses.

### PSA detection

For PSA detection, we employed antibody-modified polystyrene beads and PSA antigen (Nanopia PSA-N) acquired from Sekisui Medical Co., Ltd. The assay samples were prepared by diluting the PSA antigen reagent in 1× PBS to various concentrations. The Aipore buffer, sourced from Aipore Inc. was used for the assays. The concentration of the antibody-modified polystyrene bead reagent was adjusted to 1 × 10^9^ mL^−1^. PSA antigen samples were diluted in PBS and combined with antibody-modified nanoparticles to achieve PSA antigen concentrations ranging from 0 to 29.4 fM. The bead reagent was mixed with the assay samples and incubated for 20 min at room temperature. Subsequently, 18 μL of the sample mixture was dispensed into the trans chamber, and 15 μL of 1× PBS containing a surfactant was added to the cis chamber. The ionic current was measured at 0.1 V for a duration of 10 min, utilizing Aipore-ONE™ software^20^ (Aipore Inc.) for pulse height (*I*_p_) calculations.

### Scanning electron microscopy (SEM) observation

Field emission SEM (JSM-IT800, JEOL) was used. Two microliters of the sample to be observed was placed on a Si wafer and dried at room temperature as the observation sample.

### Optical microscopy

A *trans* chamber with a 1.2-µm-diameter solid-state pore was filled with 15 µL of 1× PBS. The *cis* chamber was filled with 15 µL or 18 µL of the sample solution. The sample was a 1× PBS solution of 1 × 10^9^ mL^−1^ polystyrene beads (4010A, Thermo-Fisher Scientific) with a diameter of 1 µm. The solid-state pore module was placed on the stage of an optical microscope (DSX1000, Olympus). The area around the solid-state pore was observed in a bright field at a magnification of 1800× using a 20× objective lens.

### Mulutiphysics model simulations

Multiphysics model simulations were performed using COMSOL Multiphysics to investigate ion currents under applied hydraulic pressure. The simulation model and set parameters are given in the Supporting Information (Fig. S6).

### Supplementary Information

Supplementary Video 1.

Supplementary Video 2.

Supplementary Information 1.

## Data Availability

The measurement data of the current–time profile of the beads obtained in this study are available on Zenodo (https://doi.org/10.5281/zenodo.4529371).

## References

[1] Branton, D. *et al.* The potential and challenges of nanopore sequencing. *Nat. Biotech.* **26**, 1146–1153 (2008). 10.1038/nbt.1495 PMC2683588 18846088

[2] Dekker, C. Solid-state nanopores. *Nat. Nanotech.* **2**, 209–215 (2007). 10.1038/nnano.2007.27 18654264

[3] Howorka, S. & Siwy, Z. Nanopore analytics: sensing of single molecules. *Chem. Soc. Rev.* **38**, 2360–2384 (2009). 19623355 10.1039/b813796j

[4] Li, J. *et al.* Ion-beam sculpting at nanometre length scales. *Nature* **412**, 166–169 (2001). 11449268 10.1038/35084037

[5] Storm, A. J., Chen, J. H., Ling, X. S., Zandbergen, H. W. & Dekker, C. Fabrication of solid-state nanopores with single-nanometre precision. *Nat. Mater.* **2**, 537–540 (2003). 12858166 10.1038/nmat941

[6] Merchant, C. A. *et al.* DNA translocation through graphene nanopores. *Nano Lett.* **10**, 2915–2921 (2010). 20698604 10.1021/nl101046t

[7] Schneider, G. F. *et al.* DNA translocation through graphene nanopores. *Nano Lett.* **10**, 3163–3167 (2010). 20608744 10.1021/nl102069z

[8] Feng, J. D. *et al.* Identification of single nucleotides in MoS_2_ nanopores. *Nat. Nanotech.* **10**, 1070–1076 (2015). 10.1038/nnano.2015.219 26389660

[9] Fologea, D. *et al.* Detecting single stranded DNA with a solid state nanopore. *Nano Lett.* **5**, 1905–1909 (2005). 16218707 10.1021/nl051199m PMC2543124

[10] Li, J. L., Gershow, M., Stein, D., Brandin, E. & Golovchenko, J. A. DNA molecules and configurations in a solid-state nanopore microscope. *Nat. Mater.* **2**, 611–615 (2003). 12942073 10.1038/nmat965

[11] Wanunu, M. *et al.* Rapid electronic detection of probe-specific microRNAs using thin nanopore sensors. *Nat. Nanotech.* **5**, 807–814 (2010). 10.1038/nnano.2010.202 20972437

[12] Zahid, O. K., Wang, F., Ruzicka, J. A., Taylor, E. W. & Hall, A. R. Sequence-specific recognition of microRNAs and other short nucleic acids with solid-state nanopores. *Nano Lett.* **16**, 2033–2039 (2016). 26824296 10.1021/acs.nanolett.6b00001 PMC5367926

[13] Talaga, D. S. & Li, J. L. Single-molecule protein unfolding in solid state nanopores. *J. Am. Chem. Soc.* **131**, 9287–9297 (2009). 19530678 10.1021/ja901088b PMC2717167

[14] Kowalczyk, S. W., Hall, A. R. & Dekker, C. Detection of local protein structures along DNA using solid-state nanopores. *Nano Lett.* **10**, 324–328 (2010). 19902919 10.1021/nl903631m

[15] Plesa, C. *et al.* Fast Translocation of proteins through solid state nanopores. *Nano Lett.* **13**, 658–663 (2013). 23343345 10.1021/nl3042678 PMC4151282

[16] Wei, R. S., Gatterdam, V., Wieneke, R., Tampe, R. & Rant, U. Stochastic sensing of proteins with receptor-modified solid-state nanopores. *Nat. Nanotech.* **7**, 257–263 (2012). 10.1038/nnano.2012.24 22406921

[17] McMullen, A., de Haan, H. W., Tang, J. X. & Stein, D. Stiff filamentous virus translocations through solid-state nanopores. *Nat. Commun.* **5**, 4171 (2014). 24932700 10.1038/ncomms5171

[18] Arima, A. *et al.* Identifying single viruses using biorecognition solid-state nanopores. *J. Am. Chem. Soc.* **140**, 16834–16841 (2018). 30475615 10.1021/jacs.8b10854

[19] Arima, A. *et al.* Selective detections of single-viruses using solid-state nanopores. *Sci. Rep.* **8**, 16305 (2018). 30390013 10.1038/s41598-018-34665-4 PMC6214978

[20] Taniguchi, M. *et al.* Combining machine learning and nanopore construction creates an artificial intelligence nanopore for coronavirus detection. *Nat. Commun.* **12**, 3726 (2021). 34140500 10.1038/s41467-021-24001-2 PMC8211865

[21] Wu, H. W. *et al.* Translocation of rigid rod-shaped virus through various solid-state nanopores. *Anal. Chem.* **88**, 2502–2510 (2016). 26790522 10.1021/acs.analchem.5b04905

[22] Tsutsui, M. *et al.* Discriminating single-bacterial shape using low-aspect-ratio pores. *Sci. Rep.* **7**, 17371 (2017). 29234023 10.1038/s41598-017-17443-6 PMC5727063

[23] Tsutsui, M. *et al.* Identification of individual bacterial cells through the intermolecular interactions with peptide-functionalized solid-state pores. *Anal. Chem.* **90**, 1511–1515 (2018). 29350898 10.1021/acs.analchem.7b04950

[24] Jia, C. P. *et al.* Nano-ELISA for highly sensitive protein detection. *Biosens. Bioelectron.* **24**, 2836–2841 (2009). 19339168 10.1016/j.bios.2009.02.024

[25] Dixit, C. K., Vashist, S. K., MacCraith, B. D. & O’Kennedy, R. Multisubstrate-compatible ELISA procedures for rapid and high-sensitivity immunoassays. *Nat. Protoc.* **6**, 439–445 (2011). 21412272 10.1038/nprot.2011.304

[26] Cheng, C. M. *et al.* Paper-based ELISA. *Angew. Chem. Int. Ed Engl.* **49**, 4771–4774 (2010). 20512830 10.1002/anie.201001005

[27] Han, A. *et al.* Label-free detection of single protein molecules and protein-protein interactions using synthetic nanopores. *Anal. Chem.* **80**, 4651–4658 (2008). 18470996 10.1021/ac7025207

[28] Ren, R. *et al.* Single-molecule binding assay using nanopores and dimeric NP conjugates. *Adv. Mater.* **33**, 2103067 (2021). 10.1002/adma.202103067 PMC11469134 34323323

[29] Venkatesan, B. M. & Bashir, R. Nanopore sensors for nucleic acid analysis. *Nat. Nanotechnol.* **6**, 615–624 (2011). 21926981 10.1038/nnano.2011.129

[30] Daiguji, H. Ion transport in nanofluidic channels. *Chem. Soc. Rev.* **39**, 901–911 (2010). 20179813 10.1039/B820556F

[31] Wen, C. Y. & Zhang, S. L. Fundamentals and potentials of solid-state nanopores: A review. *J. Phys. D Appl. Phys.* **54**, 023001 (2021). 10.1088/1361-6463/ababce

[32] Lee, K. *et al.* Recent progress in solid-state nanopores. *Adv. Mater.* **30**, 1704680 (2018). 10.1002/adma.201704680 30260506

[33] Fragasso, A., Schmid, S. & Dekker, C. Comparing current noise in biological and solid-state nanopores. *ACS Nano* **14**, 1338–1349 (2020). 32049492 10.1021/acsnano.9b09353 PMC7045697

[34] Rosenstein, J. K., Wanunu, M., Merchant, C. A., Drndic, M. & Shepard, K. L. Integrated nanopore sensing platform with sub-microsecond temporal resolution. *Nat. Methods* **9**, 487–492 (2012). 22426489 10.1038/nmeth.1932 PMC3648419

[35] Fologea, D., Uplinger, J., Thomas, B., McNabb, D. S. & Li, J. L. Slowing DNA translocation in a solid-state nanopore. *Nano Lett.* **5**, 1734–1737 (2005). 16159215 10.1021/nl051063o PMC3037730

[36] Kowalczyk, S. W., Wells, D. B., Aksimentiev, A. & Dekker, C. Slowing down DNA translocation through a nanopore in lithium chloride. *Nano Lett.* **12**, 1038–1044 (2012). 22229707 10.1021/nl204273h PMC3349906

[37] Lu, B. *et al.* Pressure-controlled motion of single polymers through solid-state nanopores. *Nano Lett.* **13**, 3048–3052 (2013). 23802688 10.1021/nl402052v PMC3864131

[38] Keyser, U. F. *et al.* Direct force measurements on DNA in a solid-state nanopore. *Nat. Phys.* **2**, 473–477 (2006). 10.1038/nphys344

[39] Davenport, M. *et al.* The role of pore geometry in single nanoparticle detection. *ACS Nano* **6**, 8366–8380 (2012). 22913710 10.1021/nn303126n

[40] Taniguchi, M., Takei, H., Tomiyasu, K., Sakamoto, O. & Naono, N. Sensing the performance of artificially intelligent nanopores developed by integrating solid-state nanopores with machine learning methods. *J. Phys. Chem. C* **126**, 12197–12209 (2022). 10.1021/acs.jpcc.2c02674

[41] Tsutsui, M. *et al.* Particle trajectory-dependent ionic current blockade in low-aspect-ratio pores. *AS Nano* **10**, 803–809 (2016). 10.1021/acsnano.5b05906 26641133

[42] Qin, Z. P., Zhe, J. A. & Wang, G. X. Effects of particle’s off-axis position, shape, orientation and entry position on resistance changes of micro coulter counting devices. *Meas. Sci. Technol.* **22**, 045804 (2011). 10.1088/0957-0233/22/4/045804

[43] Saleh, O. A. & Sohn, L. L. Correcting off-axis effects in an on-chip resistive-pulse analyzer. *Rev. Sci. Instrum.* **73**, 4396–4398 (2002). 10.1063/1.1519932

[44] Prensner, J. R., Rubin, M. A., Wei, J. T. & Chinnaiyan, A. M. Beyond PSA: The next generation of prostate cancer biomarkers. *Sci. Transl. Med.* **4**, 27rv3 (2012). 10.1126/scitranslmed.3003180 PMC3799996 22461644

[45] Duffy, M. J. Biomarkers for prostate cancer: Prostate-specific antigen and beyond. *Clin. Chem. Lab. Med.* **58**, 326–339 (2020). 31714881 10.1515/cclm-2019-0693

[46] Kim, S. H. *et al.* Large-scale femtoliter droplet array for digital counting of single biomolecules. *Lab Chip* **12**, 4986–4991 (2012). 22961607 10.1039/c2lc40632b

[47] Rissin, D. M. *et al.* Single-molecule enzyme-linked immunosorbent assay detects serum proteins at subfemtomolar concentrations. *Nat. Biotech.* **28**, 595–599 (2010). 10.1038/nbt.1641 PMC2919230 20495550

[48] Platt, M., Willmott, G. R. & Lee, G. U. Resistive pulse sensing of analyte-induced multicomponent rod aggregation using tunable pores. *Small* **8**, 2436–2444 (2012). 22570187 10.1002/smll.201200058

[49] Chuah, K. *et al.* Nanopore blockade sensors for ultrasensitive detection of proteins in complex biological samples. *Nat. Commun.* **10**, 2109 (2019). 31068594 10.1038/s41467-019-10147-7 PMC6506515

[50] Wu, Y. F., Yao, Y., Cheong, S., Tilley, R. D. & Gooding, J. J. Selectively detecting attomolar concentrations of proteins using gold lined nanopores in a nanopore blockade sensor. *Chem. Sci.* **11**, 12570–12579 (2020). 34094456 10.1039/D0SC04552G PMC8163308

[51] Liu, A., Zhao, F., Zhao, Y., Shangguan, L. & Liu, S. A portable chemiluminescence imaging immunoassay for simultaneous detection of different isoforms of prostate specific antigen in serum. *Biosens. Bioelectron.* **81**, 97–102 (2016). 26922048 10.1016/j.bios.2016.02.049

