# Supplementary material for: Immunological assay using a solid-state pore with a low limit of detection
Source: Sci Rep. 2024 Jul 19;14:16686. doi: 10.1038/s41598-024-67112-8 (PMC11271571; doi:10.1038/s41598-024-67112-8)
Supplement: Supplementary file 3 — Supplementary Information 1. [file 41598_2024_67112_MOESM3_ESM.pdf]

Supporting Information for

Immunological Assay Using a Solid-State Pore with a Low Limit of Detection

Hiroyasu Takei, Tomoko Nakada, lat Wai Leong, Atsuki Ito, Kakeru Hanada, Hinako Maeda, Muhammad Shan Sohail, Kazuhiko Tomiyasu, Osamu Sakamoto, Norihiko Naono, and Masateru Taniguchi

Supporting Information includes:

1. Table S1 Number of pulses obtained by measuring beads with solid-state pores.
2. Table S2 Number of pulses obtained by measuring beads with solid-state pores of different concentrations.
3. Table S3 Number of pulses obtained by measuring beads with solid-state pores of different concentrations.
4. Table S4 Number of pulses obtained from solid-state pore measurements of samples with different mixing ratios of beads with a diameter of 500 nm.
5. Table S5 Number of pulses obtained from solid-state pore measurements of samples with different mixing ratios of beads with diameters of 300, 500, and 600 nm.
6. Table S6 Number of pulses obtained when different concentrations of PSA were added.
7. Figure S1 Scatter plots and histograms of pulse heights and width at different mixing ratios of 500-nm-diameter beads.

8. Figure S2 Scatter plot and histograms of pulse heights and width for suspensions measured at 300 nm, 500 nm, and 600 nm mixed in equal proportions.
9. Figure S3 SEM images of polystyrene bead aggregation at different PSA concentrations.
10. Figure S4 I_p histogram and multiple Gaussian function fitting result of polystyrene bead aggregation measurement.
11. Figure S5 Measurement of pseudoclinical specimens using Adapore
12. Figure S6 Multiphysics model simulations.
13. Supporting Video 1 Optical microscopic observation under conditions of no hydraulic head pressure
14. Supporting Video 2 Optical microscopy under hydraulic head pressure conditions

Table S1 Number of pulses obtained by measuring beads with solid-state pores. These data correspond to Figure 2b–2d.

Nominal bead size (nm)	Average bead size (nm)	σ (nm)	Number of pulses
200	203	4	1048
300	303	6	1875
500	496	8	1275
600	600	9	1401
1000	1030	11	1423

Table S2 Number of pulses obtained by measuring beads with solid-state pores of different concentrations. These data correspond to Figure 2e.

Nominal bead size (nm)	Bead concentration (1/mL)	Number of pulses
300	1.6×10^7	5
		8
		11
	6.3×10^7	42
		48
		40
	2.5×10^8	170
		210
		172
	1.0×10^9	659
		635
		687
	4.0×10^9	2836
		2981
		3324
500	1.6×10^7	18
		13
		8
	6.3×10^7	59
		43
		38
	2.5×10^8	158
		190
		196

	1.0×10^9	675
		710
		668
	4.0×10^9	2548
		2365
		2234
600	1.6×10^7	16
		8
		4
	6.3×10^7	66
		44
		46
	2.5×10^8	200
		213
		230
	1.0×10^9	860
		788
		892
	4.0×10^9	3277
		1105
		2807

Table S3 Number of pulses obtained by measuring beads with solid-state pores of different concentrations. These data correspond to Figure 2f.

Nominal bead size (nm)	Bead concentration (1/mL)	Number of pulses
100	5.0×10^7	2
	5.0×10^8	14
	2.0×10^9	3
200	5.0×10^7	35
	5.0×10^8	212
	2.0×10^9	283
300	5.0×10^7	72
	5.0×10^8	865
	2.0×10^9	1120
500	5.0×10^7	38
	5.0×10^8	632
	2.0×10^9	1576
600	5.0×10^7	54
	5.0×10^8	695
	2.0×10^9	1645
1000	5.0×10^7	47
	5.0×10^8	397
	2.0×10^9	1423

Table S4 Number of pulses obtained from solid-state pore measurements of samples with different mixing ratios of beads with a diameter of 500 nm. These data correspond to Figure 3a–3c.

Mixing ratio of 500-nm-diameter beads (%)	Number of pulses
0.0	2836
	2981
	3324
1.0	2451
	2376
	2418
2.5	2805
	2593
	2166
5.0	1974
	3370
	2516
10	2884
	2955
	2387
20	2732
	3742
	3077
40	1993
	2371
	2136

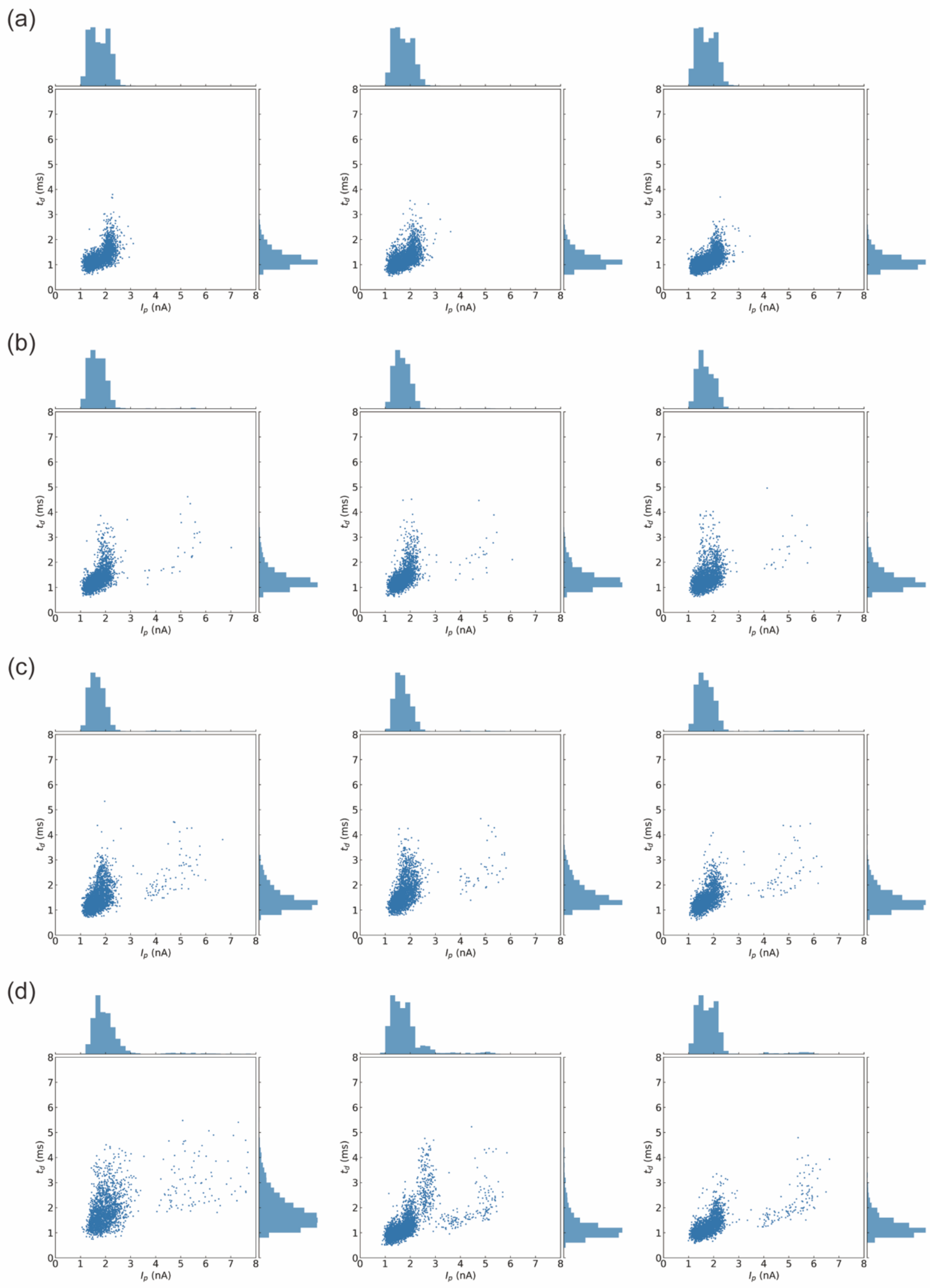
(a)
(b)
(c)
(d)
t_d (ms)
I_p (nA)

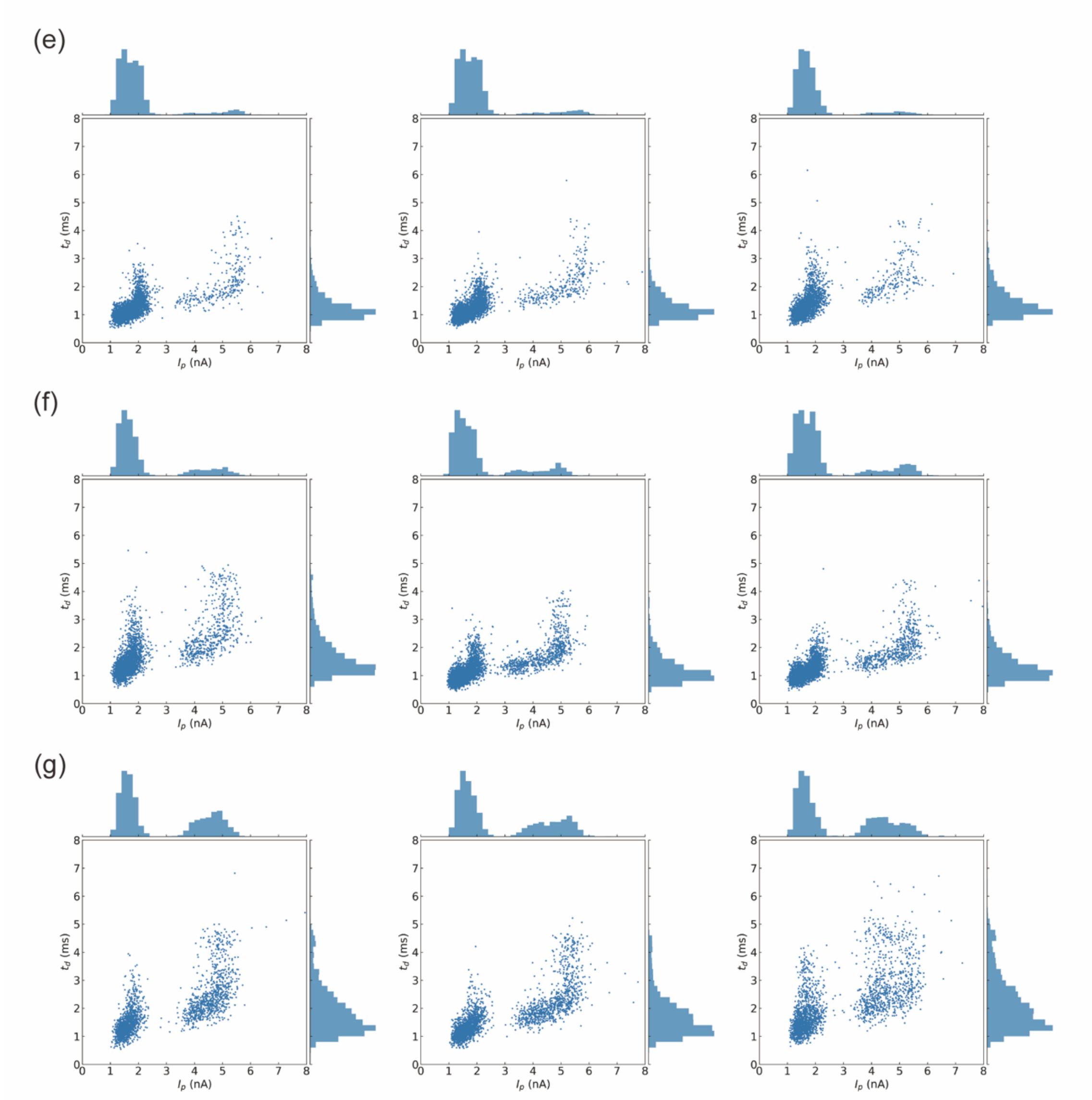

Figure S1 Scatter plots and histograms of pulse heights and width at different mixing ratios of 500 nm beads. a, 0.0%, **b**, 1.0%, **c**, 2.5%, **d**, 5.0%, **e**, 0.0%, **f**, 20.0%, and **g**, 40.0%. At least 2,000 points or more are plotted with each measurement data of 3 min or more. Under each mixing ratio condition, the measurements were conducted in triplicate.

Table S5 Number of pulses obtained from solid-state pore measurements of samples with different mixing ratios of beads with diameters of 300, 500, and 600 nm. These data correspond to Figure 4.

Mixed ratio (300 nm:500 nm:600 nm)	Number of pulses
1:3:2	1751
2:3:1	3914
3:1:2	2275
3:2:1	2376

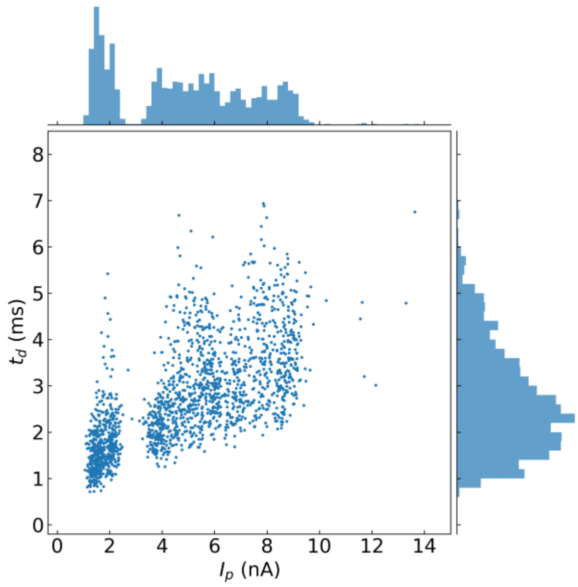

Figure S2 Scatter plot and histograms of pulse heights and width for suspensions measured at 300, 500, and 600 nm mixed in equal proportions. I_{p} and t_{d} were calculated and plotted from 1,753 waveforms acquired in one measurement.

Table S6 Number of pulses obtained when different concentrations of PSA were added. These data correspond to Figure 5.

PSA concentration (fM)	Number of pulses
0	416
	616
	441
2.94	441
	556
	360
29.4	191
	445
	389
294	196
	374
	365
2940	85
	75
	336
29400	19
	25
	92

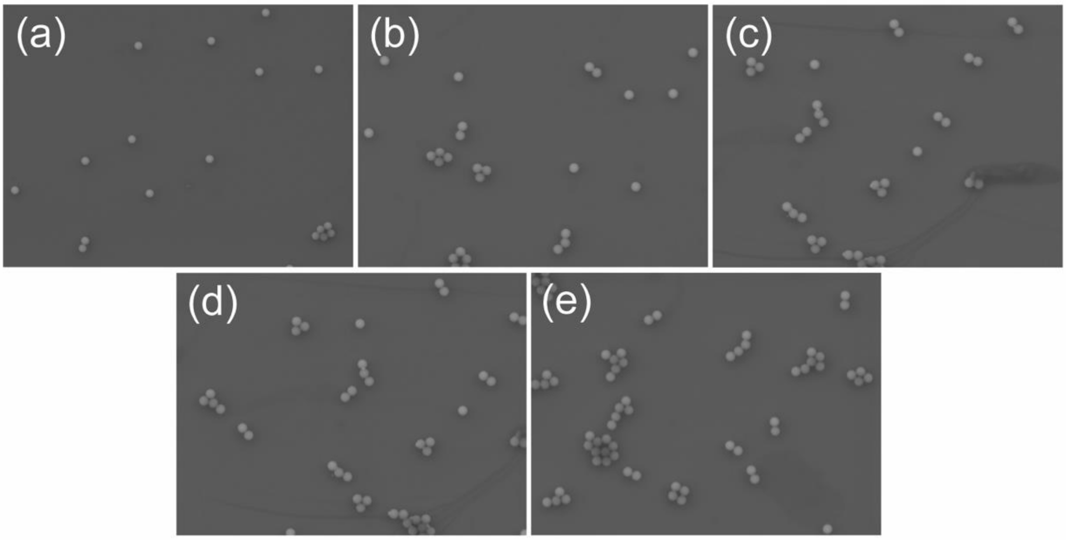

Figure S3 SEM images of polystyrene bead aggregation at different PSA concentrations. **a**, 0 fM, **b**, 29.4 fM, **c**, 0.294 pM, **d**, 2.94 pM, and **e**, 29.4 pM. The diameter of the polystyrene beads is 300 nm.

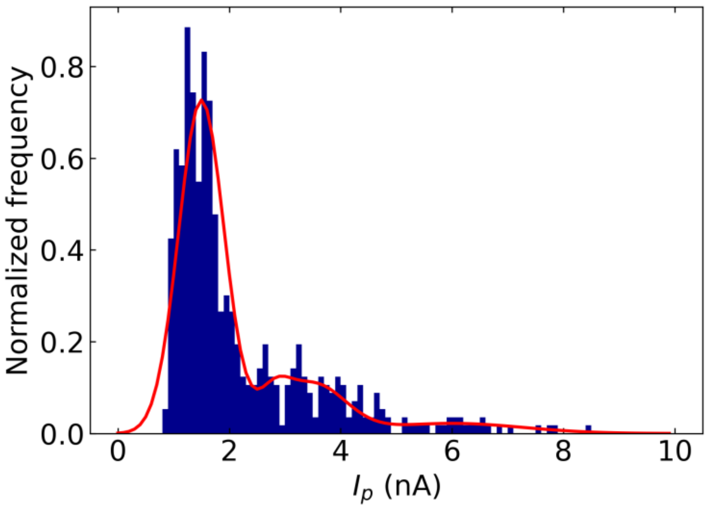

Figure S4 I_p histogram and multiple Gaussian function fitting result of polystyrene bead aggregation measurement. Gaussian fitting was performed to the measurement result of a PSA concentration of 0.294 pM by estimating the I_p distribution from the size at which aggregates could be formed. Four Gaussian distributions were used. The amplitudes were 0.73, 0.064, 0.11, and 0.022, respectively; the means were 1.5, 2.8, 3.5, and 6.0 nA, respectively; and the variances were 0.31, 0.16, 0.67, and 3.21, respectively.

Quantitative evaluation method for the degree of aggregation

In Sturges' formula, when the number of pulses is n, the number of classes k is set by the following formula, and a I_p histogram is created.

$$k = \log_2 n$$

When the frequency of the bins of the histogram is f_j and the upper and lower ends of the bins are denoted as a_j and b_j, the degree of agglutination D is defined as follows:

$$D = \frac{1}{2n} \sum_{j}^{k} f_j (a_j + b_j)$$

An analysis was conducted on five distinct samples, each with PSA concentrations of 0 pg, 1 pg, 100 pg, and 1000 pg, with three measurements per sample. Setting the aggregation threshold at 2.68 for classifying results as positive or negative—where 0 pg samples were negative and 1 pg, 100 pg, and 1000 pg samples were positive—yielded sensitivity and specificity rates of 100%.

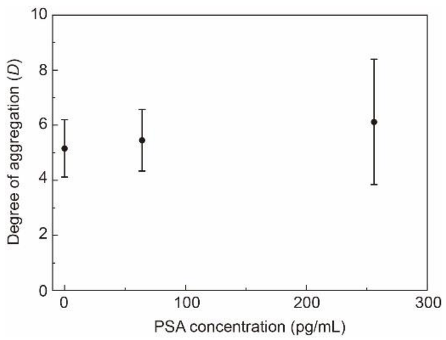

Figure S5 Measurement of pseudoclinical specimens using Adapore. Dependence of aggregation on PSA concentration. For each concentration, measurements were made using three solid-state pore modules. Error bars indicate ±2.6SD. Fetal Bovine Serum (FBS) was diluted to a 25% concentration with 1 × PBS. Pseudoclinical samples were then prepared by spiking them with 49.4 ng/mL of SEKISUI MEDICAL Nanopia PSA Calibrator to achieve final PSA concentrations of 64 pg/mL and 256 pg/mL. These prepared pseudoclinical specimens underwent filtration through a 100 nm filter (UFC30VV00, Merck). Subsequently, the filtered samples were diluted fourfold with Aipore buffer, as detailed in the Methods section. For every 100 μL of the diluted sample, 3 μL of anti-PSA antibody bead solution (Nanopia PSA, Sekisui Medical) was added. The spiked samples were incubated for 60 minutes and measured using a pore diameter of 1.2 μm.

The results, depicted in the graph below, indicate that the limit of detection could not be definitively established. This ambiguity may stem from nonspecific aggregation of the antibody beads due to contaminants, which led to an increased aggregation ratio even in the negative control samples that contained no PSA. Additionally, pore measurements were compromised by clogging, likely caused by these contaminants. To address these issues, several strategies were implemented. The concentrations of Tween and PEG in the Aipore buffer were optimized to reduce nonspecific reactions. The type and concentration of surfactants were also evaluated, with Tween-20 being replaced by Triton-X100 and KF-6011. Furthermore, the incubation time was fine-tuned, and a filter with a finer mesh than 100 nm was used for the filtration of pseudoclinical specimens. Pre-blocking of the antibody beads was conducted using Thermo's Blocking buffer, JSR's Blockmaster, or Bovine Serum Albumin (BSA) to minimize nonspecific reactions. Efforts were made to decrease the salt concentration by using 0.5 × PBS and 0.33 × PBS instead of 1 × PBS

for the pseudoclinical specimens, and by reducing the salt concentration in the Aipore buffer. Despite these extensive efforts, achieving data with a sensitivity of 1 pg/mL remained unattainable. Consequently, since the limit of detection could not be determined through the optimization of experimental conditions, the consideration to change the PSA antibody has emerged as a potential solution.

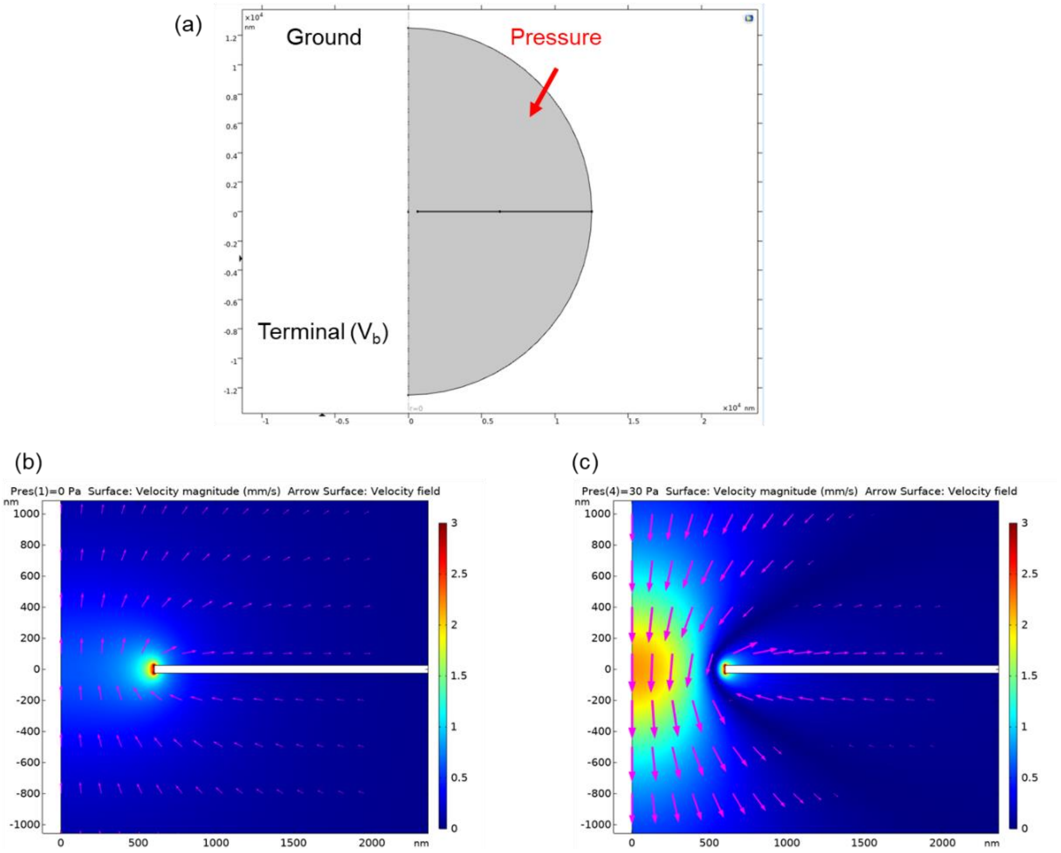

Figure S6. Model diagram and simulation results for the multiphysics model simulation. Numerical simulations of ion transport in pores are exhibited using COMSOL 5.4 with AC/DC, chemical reaction engineering, and computational fluid dynamics modules based on solving Navier−Stokes and Nernst−Planck equations under steady-state conditions. (a) Solid-state pore model structure. The diameter and thickness of the pores are 1.2 and 50 nm, respectively. The cell size of the model was 12.5 μm. The surface charge density of the silicon nitride was set to −15 mC/m^2. The solution was 1 x PBS, and $Z = 0$ was set at the center of the pore. (b) The flow velocity vector at 0.1 V with a hydraulic pressure of 0 Pa. (c) The flow velocity vector at 0.1 V with a hydraulic pressure of 30 Pa.
